# Survival analysis of patients with stage T2a and T2b perihilar cholangiocarcinoma treated with radical resection

**DOI:** 10.1186/s12885-020-07357-4

**Published:** 2020-09-03

**Authors:** Jian Zhao, Wei Zhang, Jun Zhang, Yi Zhang, Wen-Jie Ma, Si-Yun Liu, Fu-Yu Li, Bin Song

**Affiliations:** 1 Department of Radiology, West China Hospital, Sichuan University, No.37 Guoxue Alley, Wuhou District, Chengdu City, 610041 Sichuan Province P.R. China; 2 Department of Radiology, Armed Police Force Hospital of Sichuan, 614000 Leshan, Sichuan P.R. China; 3 Department of Biliary Surgery, West China Hospital, Sichuan University, Chengdu, 610041 Sichuan P.R. China; 4 GE healthcare (China), Beijing, 100176 P.R. China

**Keywords:** Perihilar cholangiocarcinoma, AJCC, Prognosis, Overall survival, Curative intent resection

## Abstract

**Background:**

Both the 7th and 8th editions of the American Joint Committee on Cancer (AJCC) staging system for perihilar cholangiocarcinoma (pCCA) had the same definition for T2a and T2b. But the value of this classification as prognostic factor remains unclear.

**Methods:**

178 patients with stage T2a or T2b who underwent curative intent resection for pCCA between Jan 2010 and Dec 2018 were enrolled. Relationships between survival and clinicopathological factors including patient demographics and tumor characteristics were evaluated using univariate and multivariate Cox regression analysis. The overall survival (OS) were calculated by Kaplan-Meier method.

**Results:**

There was no significant difference in OS between T2a and T2b groups, and the median OS duration were 37 and 31 months (*P* = 0.354). Both the 7th and 8th edition of the AJCC TNM staging demonstrated a poor prognostic predictive performance. High level of preoperative AST (≥85.0 IU/L) and CA19–9 (≥1000 U/mL), vascular resection and lower pathological differentiation of the tumor were the independent predictors for poor survival after resection.

**Conclusion:**

The newly released 8th edition of AJCC staging system demonstrated a poor ability to discriminate the prognosis of patients with stage T2a and T2b pCCA after resection.

## Background

Cholangiocarcinoma (CCA) is one of the most challenging diseases in hepatobiliary surgery field [1, 2]. CCA is a lethal epithelial malignancy of the bile duct and often presents with locally advanced or metastatic disease [3–5]. The median survival for advanced cholangiocarcinoma was less than 12 months [6]. The incidence of CCA in the U.S. continued to rise in the past 40 years [7]. In Asia, the incidence of hepatobiliary cancers was also high [8]. Because of the perihilar and distal cholangiocarcinoma had distinct epidemiology, biologic behavior, prognosis and management, the 7th edition of the American Joint Committee on Cancer (AJCC) staging system, published in 2009, further separated extrahepatic cholangiocarcinoma into two groups by either perihilar (proximal) or distal cholangiocarcinoma [9]. Therefore, the CCA have been classified into three groups anatomically: intrahepatic cholangiocarcinoma (iCCA), perihilar cholangiocarcinoma (pCCA) and distal cholangiocarcinoma (dCCA) [3, 10]. Majority (60–70%) of extrahepatic cholangiocarcinoma was pCCA [2]. pCCA was defined as cholangiocarcinoma that involve/near the biliary confluence of the right and left hepatic duct and was located to the area between the secondary branch of bile ducts and the insertion of the cystic duct into the common bile duct [4]. Radical surgery occupied the only curative option for patients with extrahepatic cholangiocarcinoma, with a 5-year survival rate ranging from 9 to 41% [11–14]. Recently, therapeutic programs were extended to liver transplantation in highly select cases [15]. Accurate stage is crucial to clinical decision-making.

Currently, the AJCC staging system remains as the most popular method in predicting survival. However, prognostic accuracy of the AJCC staging scheme is controversial. Some authors have reported that AJCC system did not predict survival accurately [16, 17]. While other investigators have noted its accuracy [18, 19]. Some researchers had proposed to change the T staging of pCCA [20, 21]. Currently, both the T2aN0M0 and T2bN0M0 are classified as TNM stage II in the 7th and 8th edition of AJCC staging system. However, in clinical practice, hemihepatectomy with resection of caudate lobe as well as an extrahepatic bile duct resection was compulsory in most surgical plans except for Bismuth type I [11]. Therefore, whether this classification (T2a and T2b) has the ability to stratify the patient’s prognosis is of great clinical value.

Thus, the objective of the current study is to validate the rationality and prognostic value of the 8th edition of AJCC staging system for stage T2a and T2b pCCA using data from a high-volume center in China.

## Methods

### Patient population

This study was performed as a retrospective observational study. The ethics committee of West China Hospital, Sichuan University approved this retrospective analysis and waived the requirement for informed consent. All patients who underwent radical surgical treatment for pCCA in our institution during January 2010 and December 2018 were identified. Routine histopathological workup was conducted for all resected pCCA by the Department of Pathology. The T stage for every patient was determined according to ‘perihilar cholangiocarcinoma’ chapter in the 8th edition of AJCC staging system. Patient selection criteria: pathologically confirmed pCCA with stage T2a and T2b. Exclusion criteria: pCCA with Tis, T1, T3, T4 tumors; patients who underwent palliative surgery or had R2 margins; primary liver tumors involved the hilum, such as iCCA, hepatocellular carcinoma (HCC) or combined HCC-cholangiocarcinoma; distal bile duct cancers; benign tumors; metastasis tumors. By these criteria, 202 patients were identified consecutively. 7 patients were excluded because N categories could not be determined. 12 patients were censored because of lost in follow-up since discharge. 5 patients died within 90 days post operation were excluded from further evaluation. Thus, a total of 178 patients were available for evaluation.

### Preoperative management and surgical procedures

All patients were evaluated with systematic inspection and elaborative imaging examination prior to surgery. Surgical procedures were finally determined and conducted according to preoperative multidisciplinary team (MDT) discussion and intraoperative exploration. Selective preoperative biliary drainage is mandatory in cholangitis and when future residual liver (FRL) is small (< 50%) in patients requiring extended resection (*n* = 32).

The operative technique consisted of complete dissection of the hilar structures, skeletonization of the hepatoduodenal ligament and removal of all the fatty and nerve tissue surrounding the common hepatic artery, the main portal vein, and the bile duct. Lymph nodes of the hepatoduodenal ligament, the proper hepatic artery and the posterior surface of the head of the pancreas were dissected routinely and retrieved. Hemihepatectomy with resection of caudate lobe was performed routinely except for Bismuth type I. The hepatectomy procedures included right and left hemi-hepatectomies, right and left trisectionectomies, and mesohepatectomy. Resection was guided by intraoperative frozen-section histology examination and intraoperative ultrasound. Roux-en-Y hepaticojejunostomy was performed meanwhile.

Patients were followed-up until January 2020. Outpatient follow-up was every 2–3 months for the first year after surgery and every 3–6 months thereafter. At each visit, assessment of liver function, measurement of tumor markers, and examination of CT and/or MRI were performed. All patients analyzed in the study had a 1 year of follow-up at least except for patient death.

### Prognostic factors collection

The admission notes, operation records, pathologic reports, and radiologic findings were reviewed for each patient. The following data were collected: demographics; operation details; hepatitis B virus (HBV) infection; cholelithiasis; fluke; percutaneous transhepatic cholangial drainage (PTCD); maximum diameter of the tumor; Bismuth type; resection margin status; vascular resection; postoperative complication; histologic grade; T stage; presence of lymph node metastasis; presence of perineural invasion; invasion of hepatic parenchyma; adjuvant therapy; preoperative total bilirubin (TBIL); direct bilirubin (DBIL); indirect bilirubin (IBIL); alanine aminotransferase (ALT); aspartate amino transferase (AST); alkaline phosphatase (ALP); gamma-glutamyl transpeptidase (GGT); carcinoembryonic antigen (CEA); carbohydrate antigen 19–9 (CA19–9); survival status. Overall survival (OS) was computed as the interval between the date of surgery and the date of death or the last follow-up. R0 was defined as no macroscopic or microscopic residual tumor. The T staging of pCCA was mainly determined by operation and pathologic records. All laboratory indicators were examined within 1 week before surgery.

### Statistical analysis

Continuous numerical data were presented as means with standard deviation or as medians with the range, and were compared by means of the student’s *t* test or Mann-Whitney U test, when appropriately. Categorical variables were compared using the χ2 test or Fisher’s exact test, when appropriately. The cutoff value of TBIL, DBIL, IBIL, ALT, AST, ALP, GGT were 157.4 μmol/L, 145.3 μmol/L, 17.8 μmol/L, 105.5 IU/L, 85.0 IU/L, 320.0 IU/L and 343.5 IU/L, which were their median respectively. The cutoff value of CEA and CA19–9 were 3.4 ng/mL (lower limit of threshold level) and 1000 U/mL (upper limit of threshold level). Survival analysis was estimated using the Kaplan-Meier method and compared using the log-rank test. Additionally, 1-, 3-and 5- year survival rates were calculated. Univariate and multivariate Cox regression analyses were performed to determine predictors of OS. Variables that were significant in univariate analysis (*P* < 0.05) were involved into the multivariate analysis. AJCC stage was not used as a dependent variable in the multivariate survival analysis to avoid confounding effect. In addition, the survival analyses were conducted for the patients stratified by stage T2a and T2b pCCA, which was further followed by the subgroup survival analyses based on different N stages of 8th edition AJCC. Variates were presented as the hazard ratio (HR) with 95% confidence interval (95%CI). The concordance index (C-index) was used to assess the performance of the 7th and the 8th editions of the AJCC staging systems [22]. Statistical analyses were performed using SPSS (version 22, IBM, Armonk, NY, USA), R software (Version: 3.5.3, *https:* *www.r-project.org*) and Medcalc (version 15.2.2, *http://www.medcalc.org*). Two tailed *P* values< 0.05 was considered to indicate a statistical difference significantly. Threshold levels of significance were adjusted for multiple comparisons by Bonferroni’s correction.

## Results

### Patient population and basic clinicopathologic characteristics

The mean age of the 178 patients was 61(range 26–80) years. The population had a male dominance (100 patients, 56.2%). The median blood loss for the resections was 400 mL (range 50–2000 mL). The median postoperative hospital stay was 17 days (range 8-50 days). Of the 178 patients, 80 were T2a and 98 were T2b. Preoperative biliary drainage was performed in 32 patients (18.0%). At the time of surgery, major hepatectomy was conducted in most patients (125, 70.2%): left hepatectomy in 60 (33.7%) patients, right hepatectomy in 35 (19.7%) patients, mesohepatectomy in 21 (11.8%) patients and a left and right trisectionectomy in 5 (2.8%) patients and 4 (2.2%) patients, respectively. The remaining 53 (29.8%) patients underwent out-hepatic bile duct resection. Caudate lobe resection was performed routinely (135, 75.8%). 10 (5.6%) patients had partial pancreatectomy. For final pathology of the resected tumor, tumor grade were classified as well- (*n* = 14, 7.9%), moderate- (*n* = 133, 74.7%) or poor- (*n* = 31, 17.4%) differentiated. Perineural invasion were present in 155 (87.1%) patients. Most patients had an R0 surgical margin (*n* = 155, 87.1%), and 23 (12.9%) patients had an R1 margin. Postoperative main complication include infection (*n* = 15), hypohepatia (*n* = 2), biliary fistula (*n* = 8), postoperative bleeding (*n* = 6), deep venous thrombosis (*n* = 2). 14 patients (7.9%) accepted gemcitabine-based chemotherapy. Lymph node metastases were present in 57 (32.0%) patients, while 121 (68.0%) patients had not metastatic lymph node identified in the surgical specimen. The median number of harvested lymph node were 5 (range 1–20).

In comparison of basic clinicopathological characteristics, age, intraoperative blood loss, caudate lobe resection and Bismuth type were significantly difference between groups T2a and T2b (Table 1). However, most of the clinicopathologic characteristics of the patients were not significantly difference.

**Table 1:** Comparison of basic clinicopathological characteristics of patients with stage T2a and T2b cholangiocarcinoma

Variable name	T2a (*n* = 80)	T2b (*n* = 98)	*P* value
Gender			0.231
Male	41	59	
Female	39	39	
Age (years)	62.0	59.0	0.018*
Maximum diameter (cm)	2.1	2.4	0.075
Intraoperative blood loss (mL)	300.0	500.0	<0.001*
TBIL (μmol/L)	175.6	153.2	0.424
DBIL (μmol/L)	153.1	136.4	0.355
IBIL (μmol/L)	17.8	17.7	0.532
ALT (IU/L)	98.5	112.0	0.807
AST (IU/L)	88.5	82.0	0.901
ALP (IU/L)	318.0	325.0	0.787
GGT (IU/L)	295.5	362.5	0.910
CA19–9 (U/mL)	184.7	283.3	0.119
CEA (ng/mL)	3.1	3.7	0.110
Cholelithiasis			0.437
With	22	22	
Without	58	76	
Preoperative biliary drainage			0.588
With	13	19	
Without	67	79	
Caudate lobe resection			<0.001*
With	44	91	
Without	36	7	
Vascular resection			0.870
With	2	4	
Without	78	94	
Perineural invasion			0.100
With	66	89	
Without	14	9	
Positive margin status			0.880
With	10	13	
Without	70	85	
Number of harvested LN			0.067
< 6	63	65	
≥ 6	17	33	
Adjuvant therapy			0.470
With	5	9	
Without	75	89	
Bismuth type			<0.001*
Type I/II	59	39	
Type III/IV	21	59	
Pathological differentiation			0.718
Well	6	8	
Moderate	62	71	
Poor	12	19	
N staging(8th edition)			0.094
N0	61	60	
N1	15	28	
N2	4	10	
Tumor stage (8th edition)			0.094
II	61	60	
IIIC	15	28	
IVA	4	10	

NOTE. *TBIL* total bilirubin; *DBIL* direct bilirubin; *IBIL* indirect bilirubin; *ALT* alanine aminotransferase; *AST* aspartate amino transferase; *ALP* alkaline phosphatase; *GGT* gamma-glutamyl transpeptidase; *CEA* carcinoembryonic antigen; *CA19–9* carbohydrate antigen 19–9; *LN* lymph node

The cutoff value of TBIL, DBIL, IBIL, ALT, AST, ALP, GGT were their median respectively. The cutoff value of CEA was the lower limit of threshold level. The cutoff value of CA19–9 was the upper limit of threshold level

* *P* value< 0.05

### Prognostic factors evaluation

During a median follow-up of 51 (range 4–117) months, 110 (61.8%) patients died. The overall median survival were 35 months. In the univariate analysis (Table S1), TBIL, AST, CA19–9, vascular resection, postoperative complication, perineural invasion, positive resection margin, pathological differentiation, N-staging and total stage were associated with poor survival. The median survival of patients with high level of TBIL (≥157.4umol/L) was 35 months, whereas that of patients with low level of TBIL (< 157.4umol/L) was 38 months (*P* = 0.025). The median survival of patients with high level of AST (≥85.0 IU/L) was 31 months, whereas that of patients with low level of AST (< 85.0 IU/L) was 41 months (*P* = 0.047). The median survival of patients with high level of CA19–9 (≥1000 U/mL) was 28 months, whereas that of patients with low level of CA19–9 (< 1000 U/mL) was 37 months (*P* = 0.015). In patients underwent vascular resection, the median survival was 10 months, while 35 months for those did not receive vascular resection (*P* = 0.008). The patients with postoperative complication had a median survival of 26 months, however, those without postoperative complication had a median survival of 36 months (*P* = 0.035). The median survival of patients without perineural invasion was 58 months, but that of patients had perineural invasion was 33 months (*P* = 0.003). The patients with positive resection margin had a median survival of 24 months, whereas those with negative resection margin had a median survival of 36 months (*P* = 0.030). The median survival for the patients with well-, moderate- and poor- differentiation tumors were 51, 37, 21 months, respectively (*P* = 0.001). The median survival of patients with stage N0, N1, N2 were 39, 27 and 28 months, respectively (*P* = 0.035).

**Fig. 1:**
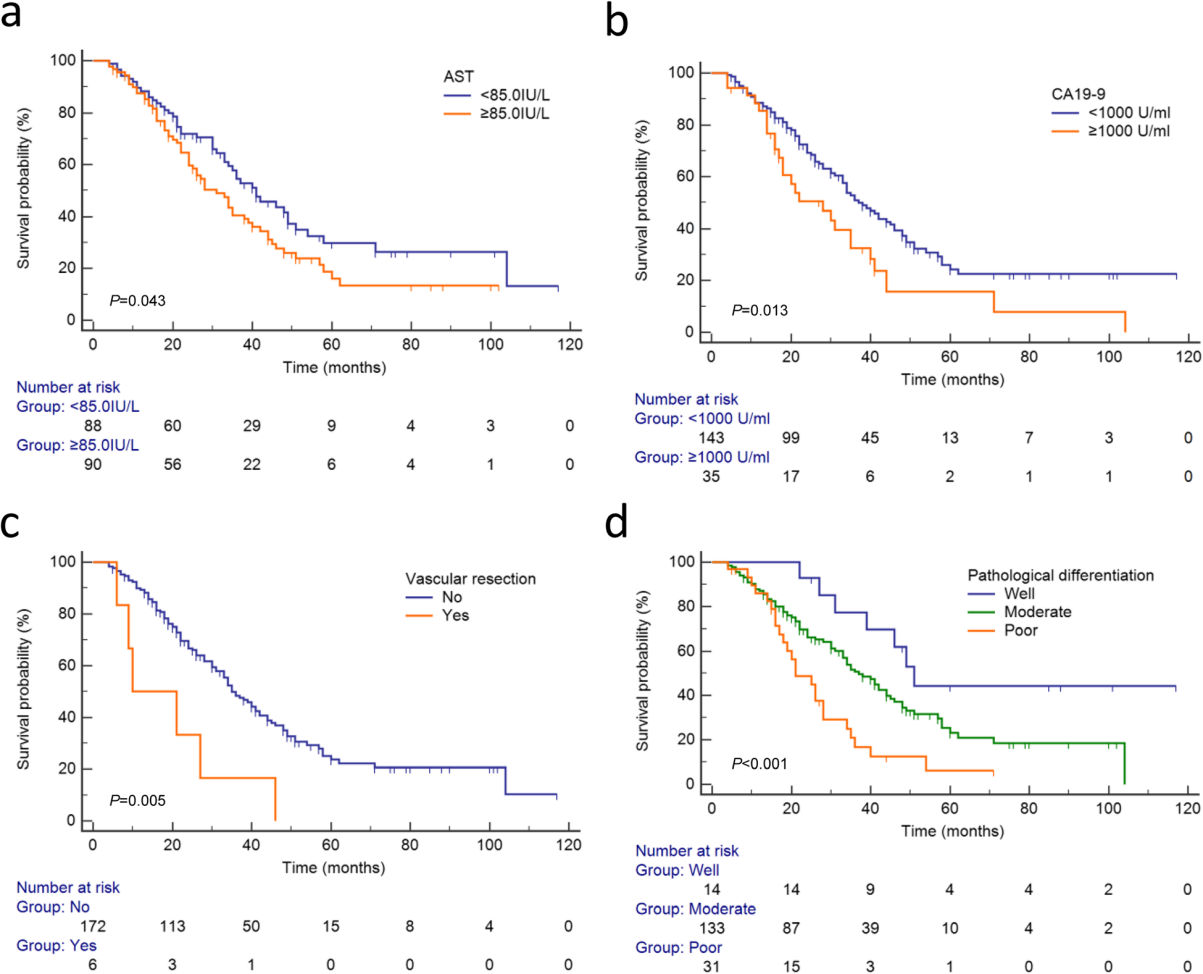
Kaplan-Meier survival curves of patients underwent surgery for pCCA. **a** Stratified by AST. **b** Stratified by CA19–9. **c** Stratified by vascular resection. (d) Stratified by pathological differentiation

To avoid collinearity of variables, total stage was not included in multivariate analysis. Thus, only high level of AST (≥85.0 IU/L), high level of CA19–9 (≥1000 U/mL), vascular resection and pathological differentiation of the tumor remained as independent predictors for poor survival (Table 2). Figure 1a-d illustrated the survival curves of patients underwent radical surgery for pCCA when stratified by AST, CA19–9, vascular resection and pathological differentiation of the tumor. The 1-, 3- and 5-year OS rates of patients with high level of AST were 87.5, 40.5 and 16.0%, respectively. Whereas patients with low level of AST had a 1-, 3- and 5-year OS rates of 88.4, 54.5 and 29.8%. Similarly, patients with high level of CA19–9 were associated with a significantly worse long-term outcome, with a 1-, 3- and 5-year OS rates of 85.4, 32.4 and 15.8%. The corresponding OS rates for patients with low level of CA19–9 was 88.6, 50.8 and 24.4% respectively. In patients who receive vascular resection, the 1- and 3- year OS rates were 50.0 and 16.7%, whereas no one survived 5 years. In contrast, patients without vascular resection had a 1-, 3- and 5-year OS rates of 89.3, 48.3 and 23.7%, respectively. The 1-, 3- and 5-year OS rates of patients with well- differentiated tumors were 100, 77.4 and 44.2%. Those of patients with moderate- differentiated tumors were 87.1, 50.7 and 23.2%. As the worst prognosis population, patients with poor- differentiated tumors had a 1-, 3- and 5-year OS rates of 86.0, 16.7 and 6.3%.

**Table 2:** Multivariate Cox regression analysis for independent prognosis factors

Variable name	HR	95.0% CI		*P* value
		Lower	Upper	
TBIL(≥157.4umol/L)	1.334	0.868	2.051	0.189
AST(≥85.0 IU/L)	1.508	1.014	2.243	0.042*
CA19–9(≥1000.0 U/mL)	1.975	1.215	3.210	0.006*
Vascular resection	3.166	1.312	7.638	0.010*
Perineural invasion	1.835	0.881	3.823	0.105
Resection margin status	1.486	0.854	2.585	0.161
Postoperative complication	1.732	0.959	3.126	0.068
Pathological differentiation				0.003*
Well	Ref.	–	–	–
Moderate	1.721	0.760	3.897	0.193
Poor	3.591	1.486	8.675	0.005*
N staging				0.605
N0	Ref.	–	–	–
N1	1.256	0.791	1.995	0.335
N2	1.166	0.568	2.396	0.676

NOTE. TBIL, total bilirubin; AST, aspartate amino transferase; CA19–9, carbohydrate antigen 19–9

The cutoff value of TBIL, AST were their median respectively; the cutoff value of CA19–9 was the upper limit of threshold level

Significant variables with *P* < 0.05 in the univariate analysis were included in the multivariate Cox PH models regression analyses

* *P* value< 0.05

### Subgroup analysis of patients with stage T2a and T2b

**Fig. 2:**
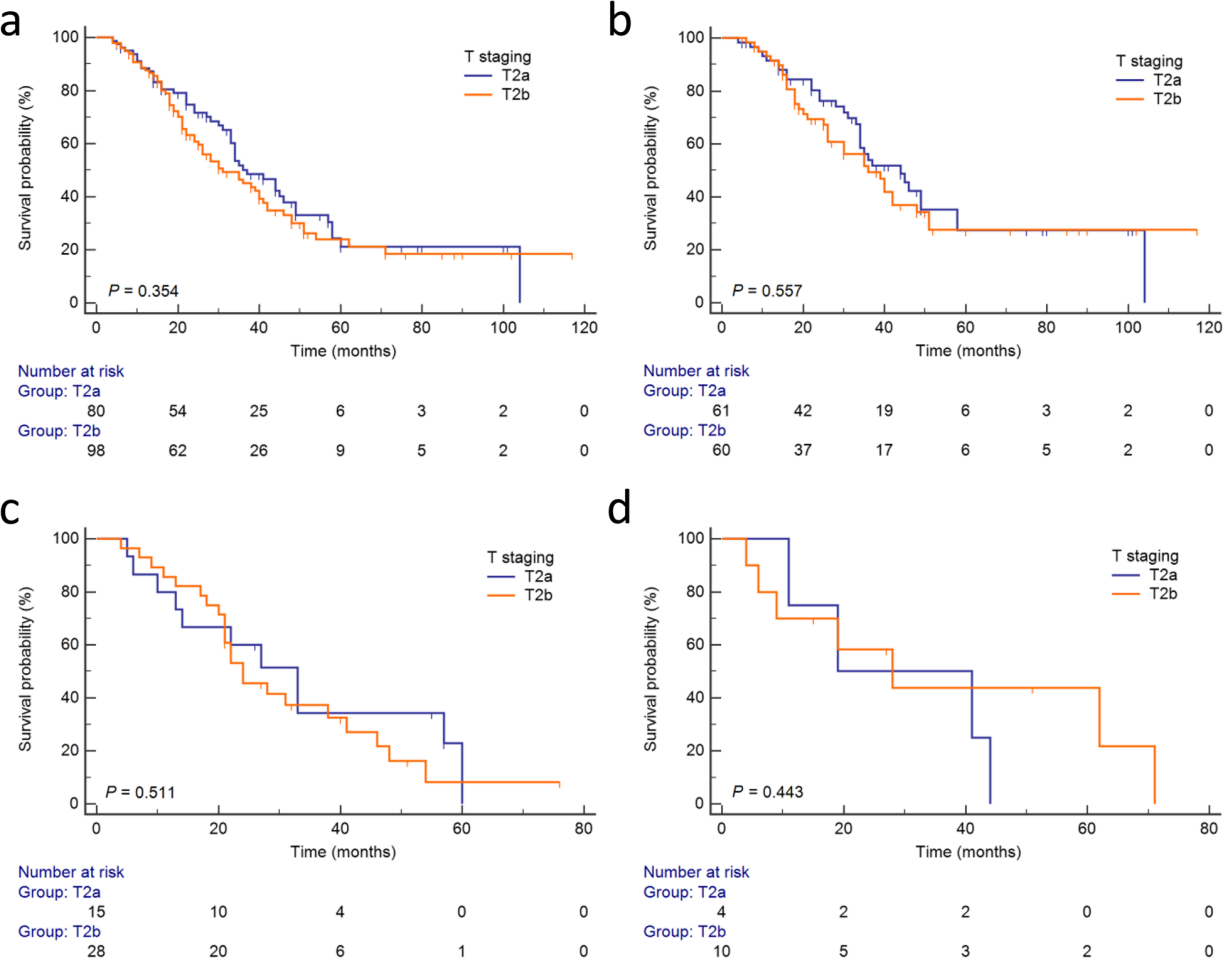
Kaplan-Meier survival curves of patients underwent surgery for pCCA. **a** Stratified by groupT2a and T2b in total cohort. **b** Stratified by groupT2a and T2b in N0 subgroup. **c** Stratified by group T2a and T2b in N1 subgroup. **d** Stratified by group T2a and T2b in N2 subgroup

In total, after curative intent resection of pCCA, there were no significant difference of survival between groups T2a and T2b (Fig. 2a, *P* = 0.354). For group T2a, the 1-, 3- and 5-year OS rates were 88.4, 50.2 and 21.3%, respectively, with a median survival of 37 months. In T2b cohort, 1-, 3- and 5-year OS rates were 87.6, 45.0 and 23.9%, respectively, with a median survival of 31 months.

Furtherly, subgroup survival analyses of patients with stage T2a and T2b were performed according to the different N stages defined by 8th edition of AJCC.

In subgroup N0, 61 patients were included in group T2a and 60 patients were categorized into group T2b. There was no significant difference of survival between group T2a and T2b as well (Fig. 2b, *P* = 0.557). For group T2a, the 1-, 3- and 5-year OS rates were 91.5, 54.0 and 27.4%, respectively, with a median survival of 44 months. In T2b cohort, 1-, 3- and 5-year OS rates were 91.4, 49.3 and 27.4%, respectively, with a median survival of 36 months.

In subgroup N1, 15 and 28 patients were respectively categorized into group T2a and T2b. No significant difference of survival existed between group T2a and T2b yet (Fig. 2c, *P* = 0.511). For group T2a, the 1-, 3- and 5-year OS rates were 80.0, 34.3 and 0.0%, respectively, with a median survival of 33 months. In T2b cohort, the 1-, 3- and 5-year OS rates were 85.7, 37.3 and 8.2%, respectively, with a median survival of 24 months.

In subgroup N2, only 14 patients were included. 4 patients belonged to group T2a and 10 patients belonged to group T2b. There was still no significant difference of survival between group T2a and T2b (Fig. 2d, *P* = 0.443). For group T2a, the 1-, 3- and 5-year OS rates were 75.0, 50.0 and 0.0%, respectively, with a median survival of 19 months. In T2b cohort, 1-, 3- and 5-year OS rates were 70.0, 43.8 and 43.8%, respectively, with a median survival of 28 months.

### Comparison of the predictive performance of the TNM staging systems in the AJCC 7th and 8th editions

**Fig. 3:**
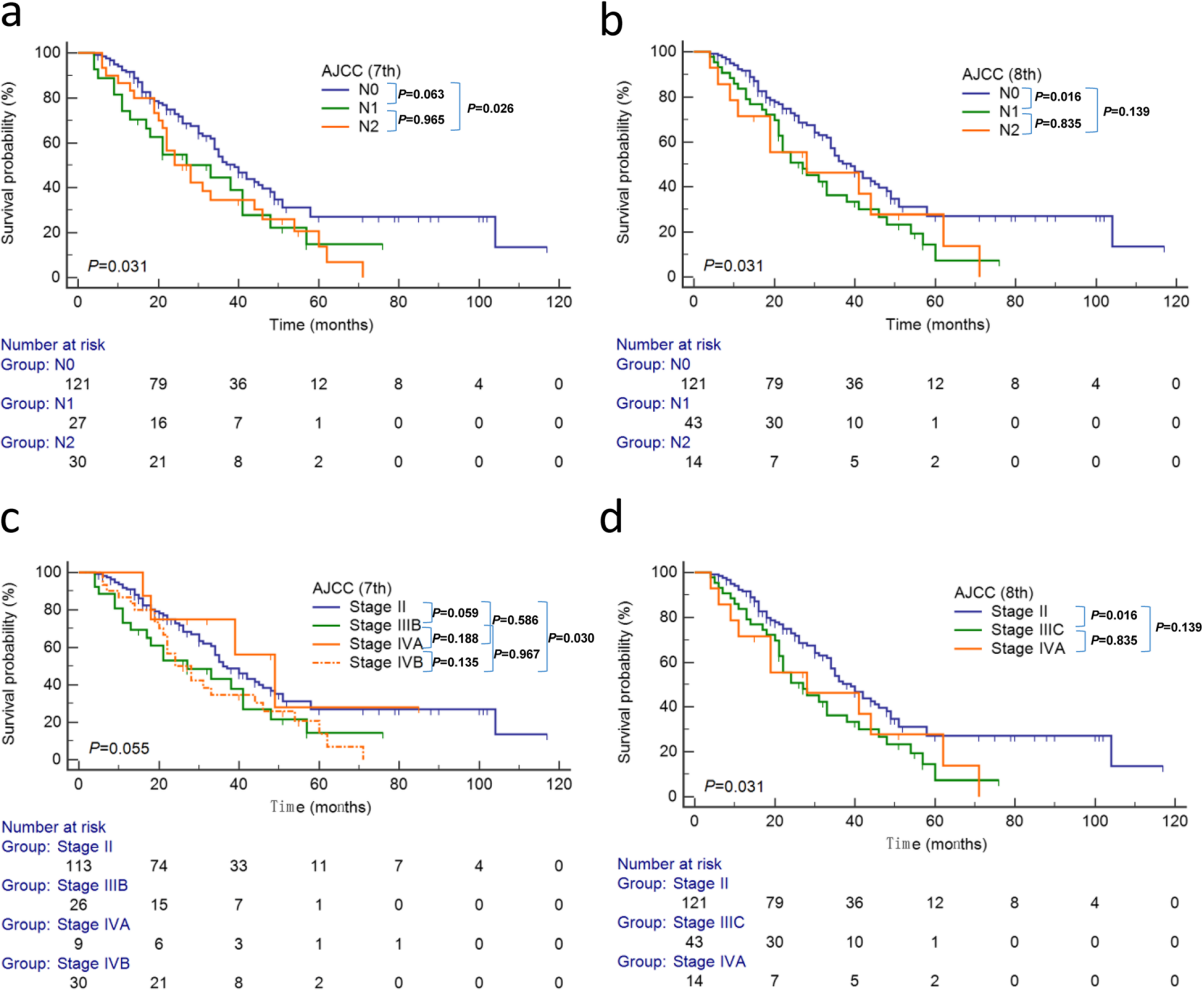
Kaplan-Meier survival curves of patients underwent surgery for pCCA. **a** Stratified by the 7th edition of the AJCC N staging. **b** Stratified by the 8th edition of the AJCC N staging. **c** Stratified by the 7th edition of the AJCC TNM staging system. **d** Stratified by the 8th edition of the AJCC TNM staging system. The overall and pairwise log-rank test results between different subgroups’ survival were interpreted as *P* values at the bottom-left and upper-right corner respectively

According to 7th edition of AJCC N staging (Fig. 3a), 121, 27 and 30 patients respectively belonged to stage N0, N1, and N2, with their median survival of 39, 33, and 24 months separately. According to the 8th edition of AJCC N staging (Fig. 3b), 121, 43 and 14 patients belonged to stage N0, N1, and N2, with a median survival of 39, 27, and 28 months, respectively.

According to 7th edition of AJCC TNM staging system, 113 patients were categorized as stage II, while 26 patients as stage IIIB, 9 patients as stage IVA, and 30 patients as stage IVB, with a median survival of 36, 27, 49 and 24 months, respectively. The 1-, 3- and 5-year OS rates were 90.9, 50.0 and 27.0% for stage II; 73.1, 43.1 and 14.4% for stage IIIB; 100, 75.0 and 28.1% for stage IVA; 86.7, 34.6 and 13.8% for stage IVB. There was no significant difference in prognosis when patients were stratified by the 7th edition of AJCC TNM staging system (Fig. 3c, *P* = 0.055).

Sorted by the 8th edition of AJCC TNM staging system, 121 patients were categorized as stage II, while 43 patients as stage IIIC and 14 patients as stage IVA, with a median survival of 39, 27 and 28 months, respectively. The 1-, 3- and 5-year OS rates of stage II were 91.4, 51.5 and 27.3%. However, those of stage IIIC were 83.7, 36.3 and 7.3%. In paired comparison, only patients with stage IIIC had a worse outcomes than those with stage II (Fig. 3d, *P* = 0.016 < 0.05/6, Bonferroni’s corrected). The 1-, 3- and 5-year OS rates were 71.4, 46.3 and 27.8% for stage IVA. Interestingly, there appeared overall significant difference between the outcome of groups categorized by the 8th edition of AJCC TNM staging system (Fig. 3d, *P* = 0.031).

The C-index for the 7th edition of the AJCC TNM staging system was 0.574 (95%CI 0.519–0.629). The C-index for the 8th edition of the AJCC TNM staging system was 0.563 (95%CI 0.512–0.614). In total, both the 7th and 8th edition of the AJCC TNM staging demonstrated a poor prognostic predictive performance (C-index < 0.7).

## Discussion

Both the 7th and 8th editions of the American Joint Committee on Cancer (AJCC) staging system for perihilar cholangiocarcinoma (pCCA) have recommended that tumor invades beyond wall of bile duct to surrounding adipose tissue could be graded as T2a and those with liver parenchyma involved into T2b. To date, there is no article focused on the prognosis of patients with stage T2a and T2b of pCCA uniquely and specifically. The current analysis represented the role of stage T2a and T2b exclusively on outcomes in resected pCCA. We found that the current AJCC T staging systems poorly stratified the prognosis of patients with T2a and T2b after curative intent resection and several clinicopathological factors of the tumor were the independent predictors for poor survival.

In comparison of basic clinicopathological characteristics between T2a and T2b groups, age, intraoperative blood loss, caudate lobe resection and Bismuth type were significantly different, whereas none of those factors were associated with poor survival, thus the baseline was balanced. There were no difference in overall survival in comparisons of group T2a and T2b, this result remained both in N0 subgroup and subgroups with lymph node metastasis (N1 and N2). This result was consistent with a few studies [23, 24]. However, another study by Kwon et al. [20] reported that the prognosis of T2b was significantly worse than T2a (*P* = 0.030). The 2-year and 3-year of survival rate were 46 and 28% for T2a, 84 and 18% for T2b in that study. Ruzzenente et al. [17] found that patients in stage T2b and T3 but not T2a and T4 had an increased risk of death compared with patients in stage T1. In their study, difference of survival between stage T2a and T2b was not discussed.

Despite improvements in treatment, pCCA was associated with limited treatment options and poor prognosis [13]. The overall median survival were 35 months in the current cohort, which was similar to the literatures [11, 18, 23]. A panel of clinicopathologic factors have been reported to influence survival of pCCA after curative intent resection [12, 25, 26]. In the current study, high level of preoperative AST (≥85.0 IU/L), high level of preoperative CA19–9 (≥1000 U/mL), vascular resection and poor differentiation of the tumor remained as independent predictors for poor survival, which were in line with previous researches [27–30].

The lymph node status have been reported as one of the most important independent prognostic predictor for patients undergoing hepatectomy for pCCA [17, 31]. In the present research, N-staging was associated with survival in the univariate analysis, while did not remain as independent predictors of poor survival in multivariate analysis, which maybe attribute to the confounding effect of tumor differentiation. Lymph node metastasis was demonstrated to correlate with tumor differentiation in other tumors [32–34]. In the cohort of our study, lymph node metastases were present in 57 (32.0%) patients which was similar to previous study [17]. Classified by the 8th edition of AJCC TNM staging system, patients with stage IIIC had a worse outcomes than those with stage II, which could more reasonably reflect the adverse effect of metastatic lymph nodes on prognosis.

T4 category excluded Bismuth type IV(bilateral second-order bile duct extension) in the 8th edition of the AJCC Staging Systems [8]. In the current study, 8 patients had bilateral second-order bile duct involved were reclassified. By reclassifying the tumors, compared to 7th edition, the 8th edition of AJCC staging system had improved ability in identifying the prognosis of the tumors at different stages (*P* = 0.031 for 8th AJCC vs. *P* = 0.055 for 7th AJCC). In a recent study, the 8th edition of AJCC staging system had a slightly better discriminatory ability with a C-index of 0.624 compared to 0.619 for the AJCC 7th edition [17]. However, predictive accuracy of the 8th edition of AJCC staging system was slightly lower than that of the 7th edition of AJCC staging system in predicting survival of pCCA with stage T2a and T2b in the current study (C-index, 0.563 vs. 0.574). Both the 7th and 8th editions of the AJCC staging systems demonstrated a poor ability in predicting prognosis of patients undergoing curative intent resection for pCCA (C-index < 0.7). Further refinements of prognostic predictors are needed to improve the predictive performance of the AJCC staging system for pCCA.

The present study had several limitations of note. Firstly, our study was limited by its retrospective nature, there may have been a selection bias in diagnosis and treatment. Secondly, only 14 patients underwent gemcitabine-based chemotherapy in this study and revealed no impact on OS. However, adjuvant therapy was demonstrated to association with improved survival, especially for those with node positive disease [35]. This result might attributed to the limit number of the patients received adjuvant therapy. Thirdly, genetic profile was not discussed in this study. It is likely that the treatment will be more and more individualized in the future when the genetic profile of a tumor can predict sensitivity or resistance to an agent. Furthermore, this cohort were collected in a single institution, enrolling a larger number of patients and multicenter cooperation are required to validate the conclusion of this study. Nevertheless, this study represent the largest cohort imploring the prognosis of pCCA with stage T2a and T2b. Lastly, this study only included a part of classification of pCCA, however, the purpose of this study was focused on the survival of stage T2a and T2b. We will make a comprehensive research considering all subtype of pCCA in the further studies.

## Conclusions

In summary, the newly released 8th edition of AJCC staging system failed to discriminate prognosis of patients with stage T2a and T2b pCCA. Both the 7th and 8th editions of the AJCC staging systems demonstrated a poor ability in predicting prognosis of patients undergoing curative intent resection for pCCA. In addition, high level of AST (≥85.0 IU/L), high level of CA19–9 (≥1000 U/mL), vascular resection and lower pathological differentiation of the tumor were the independent predictors for poor survival. At last, we proposed to merge stage T2a and T2b to simplify the AJCC staging system for pCCA in future amendments to the TNM classification.

## Supplementary information

**Additional file 1 Table S1.** Univariate regression analyses of the prognostic factors.

## Data Availability

The datasets used and analyzed during the current study are available from the corresponding author (E-mail: songlab_radiology@163.com) on reasonable request.

## Abbreviations

CCA: Cholangiocarcinoma
pCCA: Perihilar cholangiocarcinoma
OS: Overall survival
AT: Adjuvant therapy
AJCC: The American Joint Committee on Cancer
CEA: Carcinoembryonic antigen
CA19–9: Carbohydrate antigen 19–9
HR: Hazard ratio
95% CI: 95% confidence interval
