# Supplementary material for: Survival analysis of patients with stage T2a and T2b perihilar cholangiocarcinoma treated with radical resection
Source: BMC Cancer. 2020 Sep 3;20:849. doi: 10.1186/s12885-020-07357-4 (PMC7650292; doi:10.1186/s12885-020-07357-4)
Supplement: Supplementary file 1 — Additional file 1 Table S1. Univariate regression analyses of the prognostic factors. [file 12885_2020_7357_MOESM1_ESM.docx]

**Table S1.** Univariate regression analyses of the prognostic factors.

| Variable name | N | HR | 95.0% CI | | *P* value |
| --- | --- | --- | --- | --- | --- |
|  |  |  | Lower | Upper |  |
| Gender |  |  |  |  |  |
| Female | 78 | Ref. | – | – | – |
| Male | 100 | 1.371 | 0.934 | 2.014 | 0.107 |
| Age |  |  |  |  |  |
| <60years | 83 | Ref. | – | – | – |
| ≥60years | 95 | 1.066 | 0.732 | 1.553 | 0.738 |
| Maximum diameter |  |  |  |  |  |
| <3cm | 118 | Ref. | – | – | – |
| ≥3cm | 60 | 1.117 | 0.756 | 1.652 | 0.578 |
| TBIL |  |  |  |  |  |
| <157.4umol/L | 89 | Ref. | – | – | – |
| ≥157.4umol/L | 89 | 1.551 | 1.057 | 2.275 | 0.025* |
| DBIL |  |  |  |  |  |
| <145.3umol/L | 89 | Ref. | – | – | – |
| ≥145.3umol/L | 89 | 1.355 | 0.925 | 1.985 | 0.119 |
| IBIL |  |  |  |  |  |
| <17.8umol/L | 89 | Ref. | – | – | – |
| ≥17.8umol/L | 89 | 1.421 | 0.969 | 2.083 | 0.072 |
| ALT |  |  |  |  |  |
| <105.5IU/L | 89 | Ref. | – | – | – |
| ≥105.5IU/L | 89 | 1.322 | 0.905 | 1.930 | 0.149 |
| AST |  |  |  |  |  |
| <85.0IU/L | 89 | Ref. | – | – | – |
| ≥85.0IU/L | 89 | 1.471 | 1.006 | 2.150 | 0.047* |
| ALP |  |  |  |  |  |
| <320.0IU/L | 89 | Ref. | – | – | – |
| ≥320.0IU/L | 89 | 1.334 | 0.914 | 1.949 | 0.136 |
| GGT |  |  |  |  |  |
| <343.5IU/L | 89 | Ref. | – | – | – |
| ≥343.5IU/L | 89 | 1.168 | 0.802 | 1.702 | 0.418 |
| CA19-9 |  |  |  |  |  |
| <1000.0U/mL | 143 | Ref. | – | – | – |
| ≥1000.0U/mL | 35 | 1.737 | 1.112 | 2.714 | 0.015* |
| CEA |  |  |  |  |  |
| <3.4ng/mL | 92 | Ref. | – | – | – |
| ≥3.4ng/mL | 86 | 1.294 | 0.845 | 1.982 | 0.236 |
| Cholelithiasis |  |  |  |  |  |
| Without | 134 | Ref. | – | – | – |
| With | 44 | 0.968 | 0.629 | 1.489 | 0.882 |
| Preoperative biliary drainage |  |  |  |  |  |
| Without | 146 | Ref. | – | – | – |
| With | 32 | 1.155 | 0.696 | 1.916 | 0.578 |
| Vascular resection |  |  |  |  |  |
| Without | 172 | Ref. | – | – | – |
| With | 6 | 3.075 | 1.343 | 7.041 | 0.008* |
| Caudate lobe resection |  |  |  |  |  |
| Without | 43 | Ref. | – | – | – |
| With | 135 | 1.120 | 0.725 | 1.732 | 0.610 |
| Intraoperative blood loss |  |  |  |  |  |
| <400.0mL | 89 | Ref. | – | – | – |
| ≥400.0mL | 89 | 0.939 | 0.632 | 1.397 | 0.758 |
| Postoperative complication |  |  |  |  |  |
| Without | 154 | Ref. | – | – | – |
| With | 24 | 1.753 | 1.042 | 2.952 | 0.035* |
| Fluke |  |  |  |  |  |
| Without | 176 | Ref. | – | – | – |
| With | 2 | 0.622 | 0.087 | 4.466 | 0.637 |
| Perineural invasion |  |  |  |  |  |
| Without | 23 | Ref. | – | – | – |
| With | 155 | 2.848 | 1.436 | 5.650 | 0.003* |
| Positive margin status |  |  |  |  |  |
| Without | 155 | Ref. | – | – | – |
| With | 23 | 1.781 | 1.058 | 2.998 | 0.030* |
| Number of harvested LN |  |  |  |  |  |
| <6 | 128 | Ref. | – | – | – |
| ≥6 | 50 | 0.814 | 0.530 | 1.252 | 0.350 |
| Adjuvant therapy |  |  |  |  |  |
| Without | 164 | Ref. | – | – | – |
| With | 14 | 1.280 | 0.594 | 2.759 | 0.528 |
| Bismuth type |  |  |  |  |  |
| Type I/II | 98 | Ref. | – | – | – |
| Type III/IV | 80 | 1.130 | 0.771 | 1.654 | 0.531 |
| Hepatitis |  |  |  |  |  |
| Without | 48 | Ref. | – | – | – |
| With | 130 | 1.098 | 0.720 | 1.672 | 0.665 |
| Pathological differentiation |  |  |  |  | 0.001* |
| Well | 14 | Ref. | – | – | – |
| Moderate | 133 | 2.190 | 1.004 | 4.775 | 0.049 |
| Poor | 31 | 4.418 | 1.875 | 10.409 | 0.001 |
| T staging (8th edition AJCC) |  |  |  |  |  |
| T2a | 80 | Ref. | – | – | – |
| T2b | 98 | 0.838 | 0.574 | 1.223 | 0.359 |
| N staging (8th edition AJCC) |  |  |  |  | 0.035* |
| N0 | 121 | Ref. | – | – | – |
| N1 | 43 | 1.674 | 1.099 | 2.551 | 0.016 |
| N2 | 14 | 1.615 | 0.852 | 3.062 | 0.142 |
| Tumor stage (8th edition AJCC) |  |  |  |  | 0.035* |
| Ⅱ | 121 | Ref. | – | – | – |
| ⅢC | 43 | 1.674 | 1.099 | 2.551 | 0.016 |
| ⅣA | 14 | 1.615 | 0.852 | 3.062 | 0.142 |

NOTE. TBIL, total bilirubin; DBIL, direct bilirubin; IBIL, indirect bilirubin; ALT, alanine aminotransferase; AST, aspartate amino transferase; ALP, alkaline phosphatase; GGT, gamma-glutamyl transpeptidase; CEA, carcinoembryonic antigen; CA19-9, carbohydrate antigen 19-9.

The cutoff value of TBIL, DBIL, IBIL, ALT, AST, ALP and GGT were their median respectively; the cutoff value of CEA was the lower limit of threshold level; the cutoff value of CA19-9 was the upper limit of threshold level.

* *P* value<0.05
